# Pesticide residues in fruits from Riyadh markets: a three-year evaluation

**DOI:** 10.1007/s10661-025-14678-z

**Published:** 2025-10-25

**Authors:** Amjaad Ar Reshaid, Daliyah Alshemaimri, Mohamed Khabbouchi, Adel Alhotan, Saad Almutairi, Walid Aljarbou

**Affiliations:** 1 Chemistry Section, Riyadh Municipality Central Area Labs, Riyadh, Saudi Arabia; 2 Public Administration of Public Health, Riyadh Municipality, Riyadh, Saudi Arabia

**Keywords:** Contamination, Crops, Food safety, Saudi Arabia, LC–MS/MS, GC–MS/MS

## Abstract

Fruits are most commonly subjected to pesticide treatments both pre- and post-harvest, which has led to concerns regarding consumer safety. This study aimed to evaluate pesticide contamination in fruits over three years (2020 to 2022), covering a wide diversity (33 types) of fruits and a large sample size (76,290 samples). The LC–MS/MS and GC–MS/MS analyses showed that only 2.1% (1,596 samples) of fruits tested exceeded the maximum residue limits (MRLs), and only 17.8% (13,568 samples) contained detectable residues below MRLs; the majority, 80.1% (61,126 samples), were free of detectable pesticide residues or below 0.01mg/kg. Temporal trends over the three years showed that pesticide contamination levels remained relatively stable. Fungicides were the most frequently detected contaminants to exceed MRLs (53.2%; P < 0.001). Within each separate pesticide category, the most significantly and frequently detected pesticides (P < 0.001) were also identified: imazalil (17.4%) in the fungicide category, chlorpyrifos (24.2%) in the insecticide category, bifenthrin (57.5%) in the insecticide & acaricide category, ethofumesate (60.0%) in the herbicide category, and propargite (98.0%) in the acaricide category. In terms of the types of fruits tested, mandarin and buckthorn were identified as the fruits most frequently contaminated with pesticide residues below and above the MRLs, respectively. Also, lemons showed the highest frequency of pesticide contamination at elevated concentrations. These results highlight the importance of focusing on certain fruit types and pesticide categories to ensure food safety.

**Graphical Abstract:**

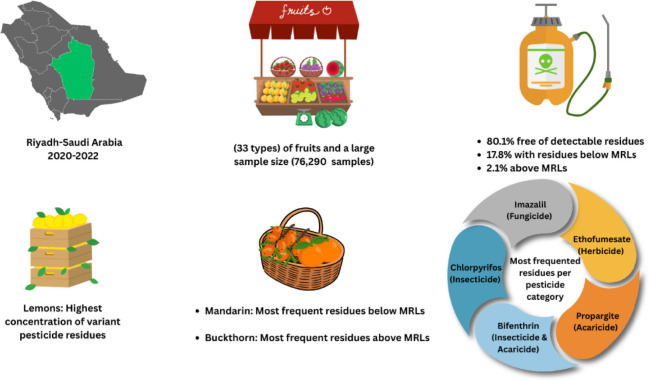

**Supplementary Information:**

The online version contains supplementary material available at 10.1007/s10661-025-14678-z.

## Introduction

The rapid growth of the global population, coupled with increasing food demand and accelerating environmental and climate changes, has led to the emergence and proliferation of new agricultural pests and diseases. These evolving challenges have reinforced the reliance on chemical pesticides, which are widely regarded as effective and economically viable tools for safeguarding crop yields (Prodhan et al., 2024). However, the misuse or excessive application of pesticides poses serious concerns for food safety, especially in fresh fruits, which are often consumed raw without any thermal processing. These concerns are further amplified by the potential application of pesticides after harvesting during various stages of production (Gelaye & Negash, 2024). The European Parliament and the Council have thus established a directive (EC) No 396/2005 to impose legal limits on residue contamination known as maximum residue levels (MRLs) (EFSA, 2024). Pesticide residues are classified into fungicides, insecticides, herbicides, acaricides, and other subgroups based on the type of harmful organism (pest) they are intended to control (European Commission, (European n.d.); Radulović et al., 2023).

Unlike other food contaminants, which may be reduced or degraded during cooking, pesticide residues tend to persist on the surface and may even penetrate the internal tissues of fruits. While rinsing with water or mild detergents can remove some surface residues, it is typically insufficient to eliminate all traces of pesticides, highlighting the critical importance of regular monitoring and strict regulatory oversight (Gelaye & Negash, 2024).

Pesticide residue contamination represents a growing concern in Saudi Arabia, due to the challenging climatic conditions characterised by high humidity and extreme temperatures. These environmental factors contribute to an increased prevalence of agricultural pests and diseases, necessitating the repeated use of multiple pesticide classes throughout the growing season (Alminderej et al., 2024). This intensive application, in turn, increases the likelihood of residue accumulation, particularly in fruits, necessitating targeted research and effective regulatory interventions to ensure consumer protection and public health. Saudi Arabia also seeks to align its food safety regulations with international standards through the oversight of agencies such as the Saudi Food and Drug Authority (SFDA), the Codex Alimentarius, the European Union (EU), and the World Health Organisation (WHO).

In recent years, a growing body of scientific literature has addressed pesticide residue levels in Saudi crops, reflecting increasing national awareness of food safety challenges (Abdallah et al., 2018; Abd-Elhaleem, 2020; Afify et al., 2022; Albedair & Alturiqi, 2021; Al-Daghri et al., 2024; Alminderej et al., 2024; Almutairi et al., 2021; Almutiriy et al., 2023; Alokail et al., 2023; Picó et al., 2021; Ramadan et al., 2020; Selim et al., 2023).

This study was conducted as a part of a comprehensive initiative launched by the Riyadh Municipality to systematically assess pesticide residues in fresh fruits in local markets. This initiative reflects the municipality’s broad commitment to strengthening food safety control systems by generating scientifically based data regarding both the presence and levels of pesticide contamination. By analysing a large and diverse sample set of commonly consumed fruits, the study aimed to identify contamination patterns, evaluate compliance with established MRLs, and provide support for evidence-based decision-making by the regulatory authorities. Ultimately, the findings are thus expected to contribute to reinforcing food safety frameworks, guiding public health policies, and increasing consumer awareness of the importance of monitoring pesticide residues in fresh produce.

## Materials and experimental methods

### Chemicals and reagents

Pesticide standards with a purity of > 98% were purchased from Restek (USA) and prepared in acetonitrile (VWR International France) at a range of concentration levels (0.01–0.2 mg/kg) for the calibration curve.

All solvents used during the experiment were HPLC-grade ≥ 99.0%, including ammonium formate, formic acid, and methanol, which were purchased from Scharlau (Barcelona, Spain). The water used was purchased from Thermo Fisher Scientific (Brussels, Belgium) with LC–MS/MS grade.

### Sample collection

Pesticide levels were evaluated among 33 different types of fresh fruits Table 1. Approximately 76,290 fruits were randomly collected from local markets in Riyadh, Saudi Arabia, over three years (2020–2022). Samples were kept in clean polyethene bags and transported to the laboratory for immediate extraction or preservation at 3℃.

### Extraction and cleanup

10 g of the homogenised sample was mixed with 10 ml of acetonitrile in a QuEChERS tube containing (4g MgSO4 + 1 g NaCl + 1 g Na3C6H5O7 + 0.5g C6H6NA2O7), vortexed for 1 min, and centrifuged at 4,000 rpm for 4 min. For cleaning up, 6 ml of supernatant was transferred into a PTFE tube containing (1200 mg MgSO4 + 400 mg PSA + 400 mg C18 + 45 mg GCB) and vortexed for 1 min, followed by centrifuging at 4000 rpm for 3 min. The supernatant was then filtered through a 0.45 μm filter into LC–MS/MS and GC–MS/MS vials.

### LC–MS/MS analysis 

Pesticide analysis was performed using an LC–MS/MS 8040 system (Shimadzu UFLC, Kyoto, Japan) consisting of a triple quadrupole mass spectrometer equipped with an electrospray ionisation (ESI) source, an LC − 30 AD pump, a CTO − 30 A column oven, a DGU-20A5R degasser, a SIL − 30 AC autosampler, and a CBM − 20 A system controller. Data acquisition and processing were carried out using LabSolutions software.

Chromatographic separation was achieved on a Shimadzu Symmetry C18 column (150 × 4.6 mm) maintained at 45 °C. Two mobile phases were used: Mobile phase A: deionised water containing 0.1% formic acid and 5 mM ammonium formate. Mobile phase B: methanol containing 0.1% formic acid and 5 mM ammonium formate. The gradient elution program was performed at a flow rate of 0.3 mL/min as follows: 90% B for 10 min, decreased to 30% B over 5 min, then to 10% B for 8 min, and finally increased back to 90% B and held for 7 min.

The autosampler was rinsed with a methanol: water (1:1, v/v) mixture before and after each injection. The injection volume was 5 µL.

The ESI interface was operated in positive and negative mode under the following parameters: interface voltage 4 kV, nebulising gas flow 3 L/min, heating gas flow 10 L/min, interface temperature 350 °C, desolvation temperature 602 °C, DL temperature 150 °C, heat block temperature 300 °C, and drying gas flow 10 L/min. The CID gas pressure was maintained at 230 kPa.

At least two multiple reaction monitoring (MRM) transitions were monitored for each analyte to ensure reliable quantification and confirmation **Supplementary Table 2**.

### GC–MS/MS analysis

Pesticides were analysed using a gas chromatography–tandem mass spectrometry system (GC–MS/MS, Shimadzu TQ8050NX, Kyoto, Japan). A capillary column RXI-5SIL ID 1651363 (30 m × 0.25 mm × 0.25 μm) was employed. The injector port was operated at 250 °C in splitless mode with an injection volume of 1 μL. Helium was used as the carrier gas at a constant flow rate of 1.6 mL/min.

The oven temperature program was set as follows: initial temperature of 90 °C held for 2 min, ramped to 130 °C at 10 °C/min, then further increased to 320 °C and held for 4 min. The total run time of the GC program was 24.33 min. The ion source temperature was set at 230 °C, and the interface temperature was maintained at 290 °C. The solvent cut time was 1.5 min. The detector voltage was adjusted to 0.6 kV.

MRM mode was employed for detection, and the relative ion intensities of selected transitions were considered to ensure accurate identification and quantification **Supplementary Table 2**.

### Statistical analysis

Data was analysed using IBM SPSS version 28. Data presented as mean percentages representing the overall temporal trend in pesticide residues (2020–2022) for free residue samples, residue samples below MRLs, and those above MRLs. The negative binomial model was used to account for the excess variance and identify significant differences across the years. A one-sample chi-square test, followed by post hoc binomial testing, was conducted to identify significantly prevalent pesticide categories and types. P-value < 0.05 was considered statistically significant.

## Results and discussion

### Validation of the analytical method

The validation method obtained for the multi-residue method in cucumber and grape matrices demonstrates good analytical performance and compliance with international guidelines (SANTE/11312/2021). Linearity was excellent for all compounds with correlation coefficients (R^2^) ≥ 0.997, confirming the reliability of the calibration models. Limits of quantification (LOQ) ranged from 0.005 to 0.1 mg/kg, which are sufficiently low to monitor pesticide residues in compliance with MRLs established by regulatory authorities. Mean recovery values generally fell within the acceptable range of 70–120%, with most compounds showing recoveries between 80–110%. Notably, compounds such as carbofuran-3-hydroxy, clothianidin, monocrotophos, and omethoate presented recoveries slightly above 110%, but these are still acceptable when considering method variability. Precision, expressed as relative standard deviation (RSD), was consistently below 15%, highlighting the robustness and repeatability of the method. Cucumber was validated for a larger number of compounds, showing stable recovery and precision across diverse pesticide categories. Grape matrices also yielded satisfactory results, with slightly higher variability for some pyrethroids (cypermethrin, deltamethrin, fenvalerate), though still within validation criteria. Overall, the method proved to be accurate, precise, and sensitive for routine monitoring of pesticide residues in fruit commodities **Supplementary Table 3**.

### Pesticide residue distribution by fruit type

**Fig. 1:**
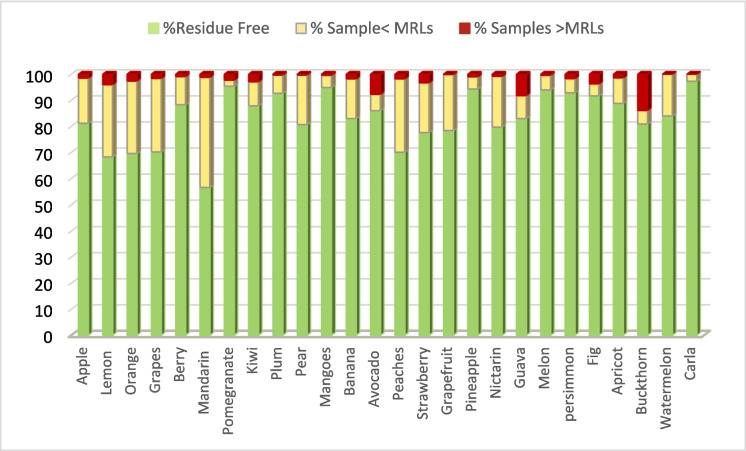
The distribution of pesticide residue levels in fruit samples

Figure 1 and Table 1 represent the distribution of pesticide residue levels across 33 fruit types based on the analysis of 76,290 samples. The results showed that 2.1% of the samples (1,596) exceeded the MRLs, while 17.8% (13,568 samples) contained detectable residues below the regulatory limits, and the majority, 80.1% (61,126 samples), were completely free of detectable pesticide residues, as their concentrations were below 0.01 mg/kg.

**Table 1:** Pesticide residue levels in fruit samples from 2020 to 2022

Fruit type	Sample size	Residue free	No. of samples < MRLs	No. of samples > MRLs
Apple	14,547	11,821 **(81.2%)**	2512 **(17.3%)**	214 **(1.5%)**
Lemon ᴬᴮ	6,389	4,361 **(68.3%)**	1769 **(27.7%)**	259 **(4%)**
Orangeᴬ	5,866	4,084 **(69.6%)**	1630 **(27.8%)**	152 **(2.6%)**
Grapesᴬ	5,667	3,979 **(70.2%)**	1599 **(28.2%)**	89 **(1.6%)**
Berry	3,803	3,357 **(88.3%)**	411 **(10.8%)**	35 **(0.9%)**
Mandarin ᴬ	3,780	2,140 **(56.6%)**	1595 **(42.2%)**	45 **(1.2%)**
Pomegranate	3,697	3,528 **(95.4%)**	86 **(2.3%)**	83 **(2.3%)**
Kiwi	3,410	2,997 **(87.9%)**	314 **(9.2%)**	99 **(2.9%)**
Plum	3,162	2,930 **(92.7%)**	223 **(7%)**	9 **(0.3%)**
Pear	3,077	2,484 **(80.7%)**	583 **(19%)**	10** (0.3%)**
Mangoes	3,051	2,895 **(94.9%)**	143 **(4.7%)**	13 **(0.4%)**
Banana	2,915	2,418 **(83%)**	442 **(15.2%)**	55 **(1.8%)**
Avocado ᴮ	2,611	2,245 **(86%)**	165 **(6.3%)**	201 **(7.7%)**
Peaches	2,283	1,600 **(70.1%)**	641 **(28.1%)**	42 **(1.8%)**
Strawberry	2,022	1,570 **(77.6%)**	386 **(19.1%)**	66 **(3.3%)**
Grapefruit	1,932	1,516 **(78.4%)**	415 **(21.5%)**	1 **(0.1%)**
Pineapple	1,770	1,669 **(94.3%)**	83 **(4.7%)**	18 **(1%)**
Nictarin	1,123	895 **(79.7%)**	219 **(19.5%)**	9 **(0.8%)**
Guava ᴮ	998	828 **(83%)**	88 **(8.8%)**	82** (8.2%)**
Melon	928	872 **(94%)**	52 **(5.6%)**	4 **(0.4%)**
persimmon	814	756 **(92.9%)**	44 **(5.4%)**	14 **(1.7%)**
Fig	722	662 **(91.7%)**	33 **(4.6%)**	27 **(3.7%)**
Apricot	717	637 **(88.8%)**	70 **(9.8%)**	10 **(1.4%)**
Buckthorn ᴮ	424	343 **(80.9%)**	22 **(5.2%)**	59 **(13.9%)**
Watermelon	256	215 **(84%)**	41 **(16%)**	0
Coconut	106	106 **(100%)**	0	0
Carla	73	71 **(97.3%)**	2 **(2.7%)**	0
Chestnut	44	44 **(100%)**	0	0
Dragon Fruit	36	36 **(100%)**	0	0
Papaya	28	28 **(100%)**	0	0
Citron	19	19 **(100%)**	0	0
Pomelo	14	14 **(100%)**	0	0
Perch	6	6 **(100%)**	0	0
	Σ 76,290	Σ 61,126	Σ 13,568	Σ 1,596
		**80.10%**	**17.80%**	**2.10%**

ᴬIndicates the four highest fruit percentages (< MRL), ᴮ Indicates the four highest fruit percentages (> MRL). The table is arranged in descending order based on the number of analysed samples

As the number of samples varied significantly across fruit types, the percentage of sample contamination was calculated separately for each fruit, relative to the total sample count, allowing for more accurate comparison.

No MRL exceedances were recorded in watermelon, coconut, carla, chestnut, dragon fruit, papaya, citron, pomelo, and perch, suggesting low exposure risk in these fruits. Apples were the most frequently tested fruit, with 14,547 total samples, and 1.5% (214 samples) exceeded the MRLs. In contrast, the highest exceedance rate was observed in buckthorn, where 13.9% of 424 samples were above the MRLs, followed by guava with 8.2% of 998 samples, avocado with 7.7% of 2611 samples, and lemon with 4% of 6389 samples exceeding the regulatory limit. These fruits were not included in earlier national surveillance studies, which had instead identified apricot, fig, and pomegranate as the most affected fruits, with 3.1% of their samples exceeding MRLs. Our findings confirmed contamination in these fruits as well, although they were not among the most heavily contaminated commodities in our dataset (Al-Daghri et al., 2024; Alokail et al., 2024).

Citrus fruits, including mandarin (42.2%), orange (27.8%), and lemon (27.7%), were among the fruits with the highest frequency of detectable pesticide residues below the MRLs. This indicates the high susceptibility of citrus fruits to pesticide application, both pre- and post-harvest (Radulović et al., 2023). Additionally, grapes showed a relatively similar rate of pesticide contamination below the MRLs (28.2%) compared to citrus fruits. This finding can be interpreted in light of data from the European Union report on pesticide residues in food, which clearly documented the widespread use of pesticides in viticulture worldwide (EFSA, 2020). These findings underscore the need for fruit-specific monitoring protocols and enhanced control measures for high-risk commodities.

### Temporal trends in pesticide residue levels (2020–2022)

**Fig. 2:**
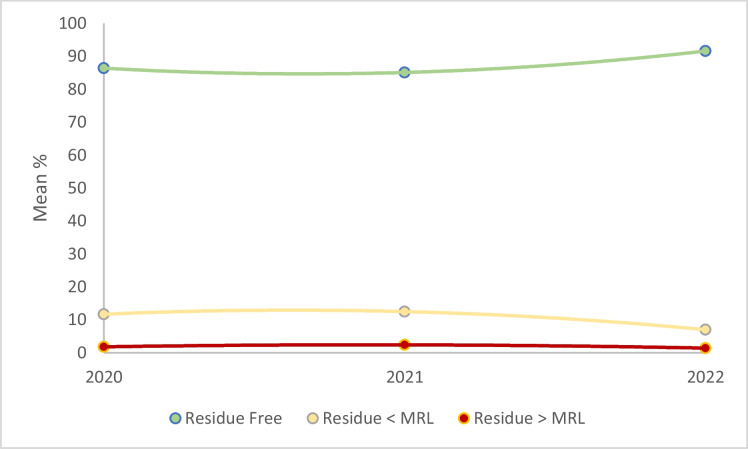
Overall temporal trend in pesticide residues (2020–2022) for residue-free, below MRL, and above MRL samples

A three-year trend analysis (2020 to 2022) was conducted to examine changes in pesticide residue patterns among various fruit types. Figure 2 illustrates the overall residue-free, below MRLs, and above MRLs samples.

The proportion of fruits with detectable residues—both below and above the MRLs—showed a slight decline over the study period: from 11.7% to 7.0% for samples below MRLs, and from 1.8% to 1.4% for samples exceeding MRLs. However, none of these changes were statistically significant (*P* > 0.05).

Interestingly, the percentage of residue-free samples showed a slight improvement, increasing from 86.4% to 91.6%. Nevertheless, this increase was also not statistically significant. These findings suggest that pesticide contamination levels were relatively stable over the three years, indicating that pesticide usage and residue control remained unchanged during the study.

On the other hand, according to the Food and Agriculture Organisation (FAO, 2023, 2024), global pesticide use increased by 4% from 2020 to 2021 and another 4% from 2021 to 2022. Also, between 1990 and 2022, pesticide use in Asia increased by 76%, highlighting a significant growth trend in agricultural chemical dependency (FAO, 2024). However, these statistics do not show the trend changes in Saudi Arabia.

### Pesticide categories and fruit contamination

**Fig. 3:**
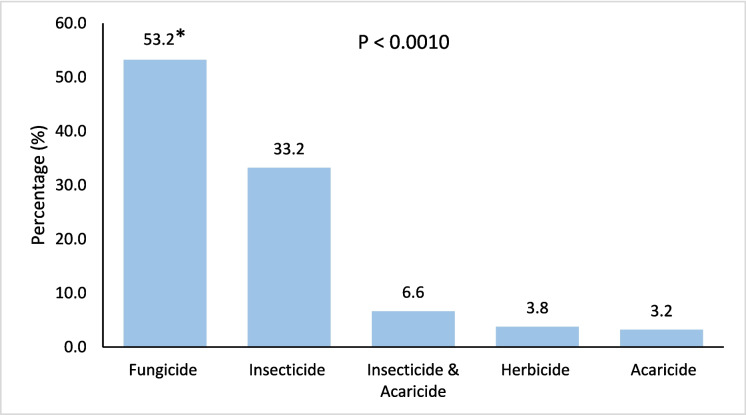
Percentage of contaminated fruit samples during 2020–2022 exceeding (> MRLs) categorised by pesticide type

Figure 3 illustrates the distribution of fruit samples with pesticide residues exceeding the MRLs, categorised by pesticide type according to the PubChem database. The results revealed that fungicides were the most frequently detected contaminants, accounting for 53.2% of all exceedances, followed by insecticides (33.2%), and insecticide & acaricide combinations (6.6%). In contrast, herbicides (3.8%) and acaricides (3.2%) represented the smallest share of violations. Statistical analysis confirmed a highly significant difference among pesticide categories (P < 0.001), suggesting that fungicides pose the highest risk of exceeding MRLs in fruit samples during the study period. Interestingly, a recent systematic review reported the same ranking of pesticide categories detected in the fruit types, except for certain categories that were not included in our study (Ahmadi et al., 2024). Moreover, that study highlighted that fungicides were detected at high concentrations in Asia, which aligns with the current findings in a way that might be attributable to environmental and geographical factors (Ahmadi et al., 2024).

Similarly, among 161 samples, 40.91% belonged to the fungicide category, representing the highest percentage, followed closely by insecticides, which also accounted for 40.91%. Although that study supports the current findings, its sample size was relatively small, and the comparisons were based on overall contamination rather than on samples exceeding the MRLs (Alokail et al., 2024). Likewise (Park et al., 2022) Analysed fruits and vegetables, detecting 32 pesticide residues, with fungicides being the most prevalent, accounting for 46.9% (15 separate pesticides).

Indeed, fungicide use is expected to increase due to climate change, the development of resistance, and the spread of invasive fungal species (Zubrod et al., 2019). A major concern is their toxicity, which may be associated with genetic damage and can affect nontarget organisms. However, their environmental impact remains understudied compared to other pesticide categories, such as insecticides and herbicides (Tisza et al., 2025; Zubrod et al., 2019).

**Fig. 4:**
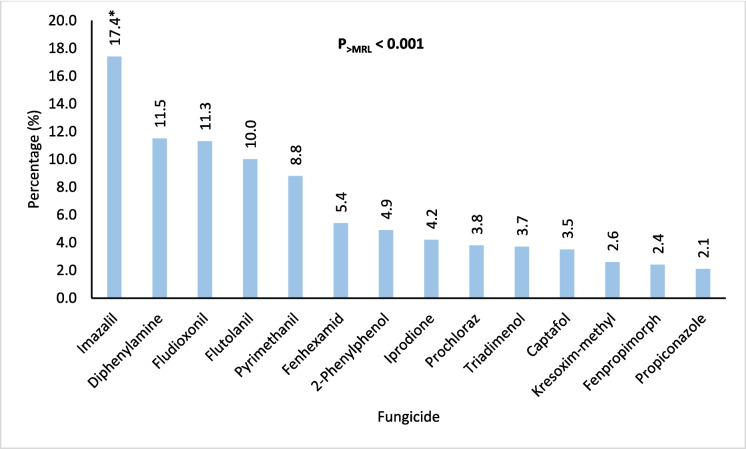
Percentage of the most contaminating pesticide exceeding (> MRLs) in the fungicide category

**Fig. 5:**
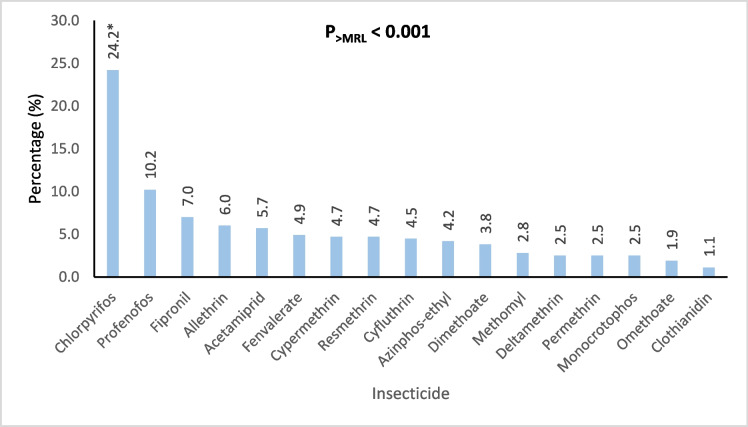
Percentage of the most contaminating pesticide exceeding (> MRLs) in the insecticide category

**Fig. 6:**
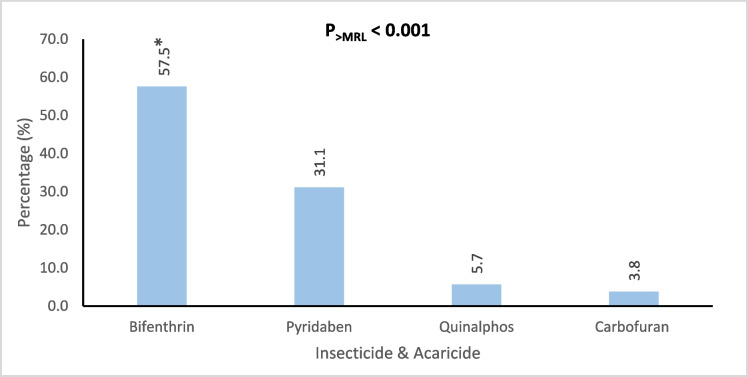
Percentage of the most contaminating pesticide exceeding (> MRLs) in the insecticide & acaricide category

**Fig. 7:**
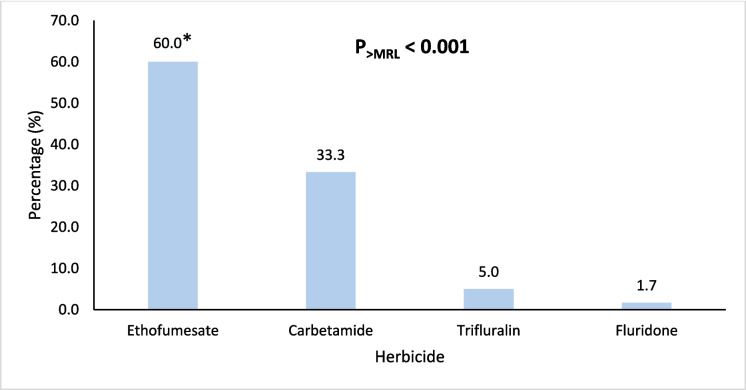
Percentage of the most contaminating pesticide exceeding (> MRLs) in the herbicide category

**Fig. 8:**
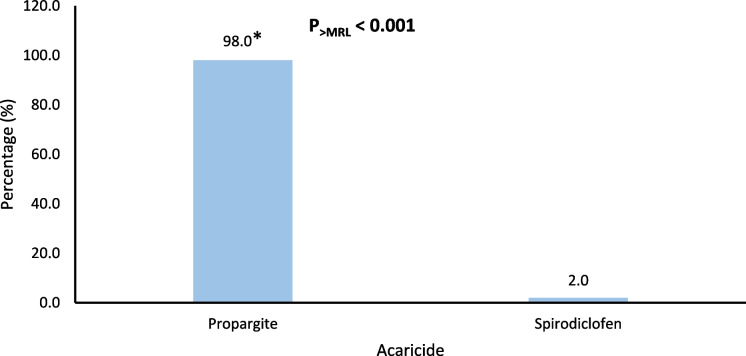
Percentage of the most contaminating pesticide exceeding (> MRLs) in the acaricide category

Figures 4, 5, 6, 7, and 8 further highlight the most frequently detected pesticides exceeding the MRLs within each category. Within each pesticide category, the most significantly and frequently detected pesticides with (P < 0.001) were as follows: imazalil (17.4%) in the fungicide category, chlorpyrifos (24.2%) in the insecticide category, bifenthrin (57.5%) in the insecticide & acaricide category, ethofumesate (60.0%) in the herbicide category, and propargite (98.0%) in the acaricide category.

According to the WHO's, 2020 classification of pesticide toxicity, imazalil, chlorpyrifos, bifenthrin, and propargite are classified as Class II (moderately hazardous), while ethofumesate is classified under Class U (unlikely to present acute hazard) (WHO, 2020). On the other hand, those pesticide residues raise concern, as imazalil and propargite are both banned, while chlorpyrifos and bifenthrin are restricted based on the decision of the Council of Ministers No. (136,618/1135/1443) in Saudi Arabia (Ministry of Environment Water & Agriculture, 2022; SFDA, 2019).

One of the challenges faced in assessing pesticide risks is the variation in banning and restriction policies across countries, despite the recognised toxicity of the mentioned pesticides (Radulović et al., 2023). This inconsistency complicates the development of definitive judgments about their safety. For instance, chlorpyrifos is banned in the EU, the USA, and Denmark, yet remains permitted in other countries, including Saudi Arabia (Foong et al., 2020). Similarly, imazalil is banned in Saudi Arabia but has been allowed elsewhere, including in the European Food Safety Authority (EFSA et al., 2023; Ministry of Environment Water & Agriculture, 2022; SFDA, 2019).

In terms of imazalil, one of the primary concerns is the potential genotoxicity and carcinogenicity of its metabolites. Also, EFSA has not yet derived MRL proposals for post-harvest uses on crops because the toxicological assessment of the metabolite R014821 is unfinalized (EFSA, 2018; EPA, 2005; Tisza et al., 2025).

A study conducted on fruits sold in Riyadh, Saudi Arabia, reported that imazalil was the most commonly detected pesticide residue (10.56%); however, its levels did not exceed the MRLs (Alokail et al., 2024). Other studies have similarly revealed that imazalil is the most frequently detected pesticide residue in citrus fruits (Fernández et al., 2001; Jurak et al., 2021; Radulović et al., 2023).

Chlorpyrifos might be expected to be the most frequently detected insecticide, as it is widely used due to its broad-spectrum toxicity against various insect species. It is also the most studied pesticide with extensive studies on human health and environmental impacts, resulting in increased regulatory restrictions and decreased use in most developed countries (DuTeaux & Koshlukova, 2024). Nevertheless, a meta-analysis study highlighted that chlorpyrifos was still the most commonly detected insecticide in fruit (W. Li et al., 2023). Similarly, (T. M. Osaili et al., 2022) detected chlorpyrifos exceeding the MRLs with the highest frequency among fruits (Osaili et al., 2022). This study is of particular interest due to its geographical proximity to our study area. Moreover, similar reversals in different countries, including China, Iran, India, Ghana, and Thailand, have supported the implication of chlorpyrifos contamination in fruit when exceeding the MRLs’ limits (Akoto et al., 2015; Essumang et al., 2013; Hadian et al., 2019; Z. Li et al., 2022; Narenderan et al., 2019; Okoffo et al., 2017; Rai et al., 2016; Sapbamrer & Hongsibsong, 2014; Taghizadeh et al., 2021; Wang et al., 2013).

### The highest residue concentrations among fruit samples

The comparison of fruit samples from 2020 to 2022 with contamination levels exceeding the MRLs revealed that lemons exhibited the highest concentration levels across multiple pesticide categories Table 2.

**Table 2:** The Highest Residue Concentrations Among Fruit Samples Exceeding MRLs (2020–2022) by Pesticide Category

Pesticide Category	Pesticide with Maximum Concentration	Maximum Concentration (mg/kg)	Fruit
Herbicide	Ethofumesate	17.6	Lemon
Fungicide	Imazalil	8.0	Lemon
Insecticide	Cypermethrin	5.0	Pear
Insecticide & Acaricide	Pyridaben	2.8	Lemon
Acaricide	Propargite	1.7	Peaches

Lemons had the highest concentrations of residues within the herbicide (ethofumesate, 17.6 mg/kg), fungicide (imazalil, 8.0 mg/kg), and insecticide & acaricide (pyridaben, 2.8 mg/kg) categories. Pears showed the highest concentration among the insecticide category (cypermethrin, 5.0 mg/kg), while peaches had the highest concentration among acaricides (propargite, 1.7 mg/kg). These findings highlight a lemon’s vulnerability to multi-category pesticide accumulation and its susceptibility to being contaminated with high pesticide concentrations.

A lemon belongs to the citrus fruit group, which is commonly treated with multiple pesticides both pre- and post-harvest. Citrus fruits are thus often considered among the most pesticide-contaminated food products (Ortelli et al., 2005; Radulović et al., 2023). Although ethofumesate belongs to Class U (unlikely to present an acute hazard), exceeding the MRLs by approximately 500 times still raises significant concern. This is also important in terms of considering the potential for chronic exposure and the implications of non-compliance with regulatory standards, which remain critical aspects of food safety assessment (WHO, 2020).

Indeed, this study has some limitations that should be acknowledged. Since the fruit samples were collected directly from markets, it was not possible to identify their source farms or assess the agricultural practices used by the farmers. In addition, it remains unclear whether the samples were locally produced or imported, which limits the ability to provide a comprehensive reflection of the crop status in Saudi Arabia. Moreover, the small sample size for some fruit types, compared to others with thousands of samples, may obscure the overall contamination pattern among fruit types.

## Conclusion

Mandarin and buckthorn were the fruits most frequently contaminated with pesticide residues below and above the MRLs, respectively. Lemons exhibited the highest concentrations of multiple pesticide residue types, which may indicate improper agricultural practices in their cultivation. Among the pesticide categories, fungicides accounted for the highest proportion of samples exceeding the MRLs. The three-year trend analysis showed no significant changes, suggesting that pesticide usage practices and residue control remained largely consistent during the study period. These findings highlight the need for continued periodic monitoring to ensure and maintain food safety, with attention to specific fruit types and pesticide categories.

## Supplementary Information

Below is the link to the electronic supplementary material.

ESM 1 (DOCX 116 KB)

## Data Availability

All data supporting the findings of this study are included in the article in the form of tables and figures. Additional details may be available from the corresponding author upon reasonable request.

## Declarations

### Clinical trial number

Clinical trial number is not applicable.
