# Supplementary material for: Pesticide residues in fruits from Riyadh markets: a three-year evaluation
Source: Environ Monit Assess. 2025 Oct 25;197(11):1253. doi: 10.1007/s10661-025-14678-z (PMC12553580; doi:10.1007/s10661-025-14678-z)
Supplement: Supplementary file 1 — (DOCX 116 KB) [file 10661_2025_14678_MOESM1_ESM.docx]

| **Supplementary Table 1. Descriptive statistics according to Year** | | | | | | | | | | | | | | | | | | | | |
| --- | --- | --- | --- | --- | --- | --- | --- | --- | --- | --- | --- | --- | --- | --- | --- | --- | --- | --- | --- | --- |
|  | **Pesticide Type** | | | | **Residue Free** | | | | **With Residue** | | | | **Residue < MRL** | | | | **Residue > MRL** | | | |
|  | **2020** | **2021** | **2022** | **P-value** | **2020** | **2021** | **2022** | **P-value** | **2020** | **2021** | **2022** | **P-value** | **2020** | **2021** | **2022** | **P-value** | **2020** | **2021** | **2022** | **P-value** |
| **Overall** | 19.4 ± 3.0 | 16.0 ± 2.6 | 12.4 ± 2.1 | 0.10 | 86.4 ± 2.1 | 85.1 ± 2.3 | 91.6 ± 1.4 | 0.96 | 13.5 ± 2.1 | 14.9 ± 2.3 | 8.4 ± 1.4 | 0.13 | 11.7 ± 2.0 | 12.5 ± 2.2 | 7.0 ± 1.3 | 0.12 | 1.8 ± 0.4 | 2.4 ± 0.7 | 1.4 ± 0.5 | 0.09 |
| **Apple** | 63 | 52 | 37 | 0.96 | 5713 (79.5) | 3626 (79.8) | 2482 (88) | 1.00 | 1470 (20.5) | 918 (20.2) | 338 (12) | 0.32 | 1358 (18.9) | 837 (18.4) | 317 (11.2) | 0.92 | 112 (1.6) | 81 (1.8) | 21 (0.7) | 0.81 |
| **Apricot** | 12 | 10 | 7 | 0.90 | 322 (87.3) | 211 (87.9) | 104 (96.3) | 1.00 | 47 (12.7) | 29 (12.1) | 4 (3.7) | 0.29 | 42 (11.4) | 25 (10.4) | 3 (2.8) | 0.60 | 5 (1.4) | 4 (1.7) | 1 (0.9) | 0.95 |
| **Avocado** | 25 | 24 | 7 | 0.88 | 1110 (85.6) | 659 (80.5) | 476 (96) | 0.99 | 186 (14.4) | 160 (19.5) | 20 (4) | 0.55 | 83 (6.4) | 78 (9.5) | 4 (0.8) | 0.22 | 103 (7.9) | 82 (10) | 16 (3.2) | 0.71 |
| **Banana** | 26 | 25 | 15 | 0.91 | 1225 (82.1) | 785 (80.9) | 408 (90.1) | 1.00 | 267 (17.9) | 185 (19.1) | 45 (9.9) | 0.41 | 238 (16.0) | 166 (17.1) | 38 (8.4) | 0.86 | 29 (1.9) | 19 (2) | 7 (1.5) | 0.98 |
| **Berry** | 25 | 26 | 20 | 0.92 | 1585 (88) | 971 (85.8) | 801 (92.2) | 1.00 | 217 (12) | 161 (14.2) | 68 (7.8) | 0.55 | 201 (11.2) | 147 (13) | 63 (7.2) | 0.91 | 16 (0.9) | 14 (1.2) | 5 (0.6) | 0.88 |
| **Buckthorn** | 9 | 5 | 6 | 0.54 | 185 (83.7) | 128 (78.5) | 30 (75) | 1.00 | 36 (16.3) | 35 (21.5) | 10 (25) | 0.07 | 14 (6.3) | 4 (2.5) | 4 (10) | 0.66 | 22 (10) | 31 (19) | 6 (15) | 0.90 |
| **Carla** | 1 | 1 | 0 | 0.80 | 40 (97.6) | 24 (96) | 7 (100) | 1.00 | 1 (2.4) | 1 (4) | 0 (0) | 0.59 | 1 (2.4) | 1 (4) | 0 (0) | 0.80 | 0 (0) | 0 (0) | 0 (0) | -- |
| **Chestnut** | 0 | 0 | 0 | -- | 22 (100) | 11 (100) | 11 (100) | 1.00 | 0 (0) | 0 (0) | 0 (0) | -- | 0 (0.0) | 0 (0) | 0 (0) | -- | 0 (0) | 0 (0) | 0 (0) | -- |
| **Citron** | 0 | 0 | 0 | -- | 11 (100) | 7 (100) | 1 (100) | 1.00 | 0 (0) | 0 (0) | 0 (0) | -- | 0 (0.0) | 0 (0) | 0 (0) | -- | 0 (0) | 0 (0) | 0 (0) | -- |
| **Coconut** | 0 | 0 | 0 | -- | 52 (100) | 4 (100) | 50 (100) | 1.00 | 0 (0) | 0 (0) | 0 (0) | -- | 0 (0.0) | 0 (0) | 0 (0) | -- | 0 (0) | 0 (0) | 0 (0) | -- |
| **Dragon Fruit** | 0 | 0 | 0 | -- | 17 (100) | 14 (100) | 5 (100) | 1.00 | 0 (0) | 0 (0) | 0 (0) | -- | 0 (0.0) | 0 (0) | 0 (0) | -- | 0 (0) | 0 (0) | 0 (0) | -- |
| **Fig** | 10 | 9 | 3 | 0.97 | 285 (89.9) | 304 (92.4) | 73 (96.1) | 1.00 | 32 (10.1) | 25 (7.6) | 3 (3.9) | 0.25 | 18 (5.7) | 13 (4.0) | 2 (2.6) | 0.89 | 14 (4.4) | 12 (3.6) | 1 (1.3) | 0.78 |
| **Grapefruit** | 20 | 19 | 16 | 0.92 | 741 (78.5) | 410 (73.5) | 365 (84.9) | 0.99 | 203 (21.5) | 148 (26.5) | 65 (15.1) | 0.62 | 203 (21.5) | 148 (26.5) | 64 (14.9) | 0.92 | 0 (0) | 0 (0) | 1 (0.2) | 0.31 |
| **Grapes** | 49 | 44 | 38 | 0.86 | 1881 (66.7) | 1221 (66.2) | 877 (87.6) | 0.97 | 941 (33.3) | 623 (33.8) | 124 (12.4) | 0.35 | 903 (32.0) | 584 (31.7) | 112 (11.2) | 0.70 | 38 (1.3) | 39 (2.1) | 12 (1.2) | 0.92 |
| **Guava** | 14 | 11 | 11 | 0.81 | 431 (84.2) | 262 (81.1) | 135 (82.8) | 1.00 | 81 (15.8) | 61 (18.9) | 28 (17.2) | 0.71 | 45 (8.8) | 27 (8.4) | 16 (9.8) | 0.99 | 36 (7) | 34 (10.5) | 12 (7.4) | 0.95 |
| **Kiwi** | 17 | 14 | 15 | 0.82 | 1471 (87.9) | 972 (86.2) | 554 (91.1) | 1.00 | 203 (12.1) | 156 (13.8) | 54 (8.9) | 0.55 | 156 (9.3) | 115 (10.2) | 43 (7.1) | 0.96 | 47 (2.8) | 41 (3.6) | 11 (1.8) | 0.89 |
| **Lemon** | 55 | 45 | 36 | 0.87 | 2087 (63.8) | 1369 (65.4) | 905 (88.3) | 0.97 | 1185 (36.2) | 723 (34.6) | 120 (11.7) | 0.30 | 1058 (32.3) | 619 (29.6) | 92 (9) | 0.60 | 127 (3.9) | 104 (5) | 28 (2.7) | 0.92 |
| **Mandarin** | 43 | 35 | 29 | 0.80 | 1094 (56.3) | 674 (51.5) | 372 (70.5) | 0.97 | 850 (43.7) | 634 (48.5) | 156 (29.5) | 0.49 | 826 (42.5) | 615 (47) | 154 (29.2) | 0.94 | 24 (1.2) | 19 (1.5) | 2 (0.4) | 0.67 |
| **Mangoes** | 13 | 11 | 12 | 0.83 | 1437 (94.7) | 899 (94.4) | 559 (96) | 1.00 | 80 (5.3) | 53 (5.6) | 23 (4) | 0.44 | 75 (4.9) | 48 (5) | 20 (3.4) | 0.96 | 5 (0.3) | 5 (0.5) | 3 (0.5) | 0.94 |
| **Melon** | 17 | 13 | 6 | 0.98 | 468 (93.4) | 276 (94.2) | 128 (95.5) | 1.00 | 33 (6.6) | 17 (5.8) | 6 (4.5) | 0.09 | 32 (6.4) | 16 (5.5) | 4 (3) | 0.87 | 1 (0.2) | 1 (0.3) | 2 (1.5) | 0.53 |
| **Nictarin** | 19 | 14 | 24 | 0.86 | 421 (79.4) | 228 (80) | 246 (79.9) | 1.00 | 109 (20.6) | 57 (20) | 62 (20.1) | 0.47 | 104 (19.6) | 54 (18.9) | 61 (19.8) | 1.00 | 5 (0.9) | 3 (1.1) | 1 (0.3) | 0.79 |
| **Orange** | 47 | 38 | 28 | 0.92 | 2063 (69.5) | 1170 (61.4) | 851 (86) | 0.97 | 906 (30.5) | 737 (38.6) | 139 (14) | 0.54 | 823 (27.7) | 672 (35.2) | 135 (13.6) | 0.78 | 83 (2.8) | 65 (3.4) | 4 (0.4) | 0.31 |
| **Papaya** | 0 | 0 | 0 |  | 12 (100) | 11 (100) | 5 (100) | 1.00 | 0 (0) | 0 (0) | 0 (0) | -- | 0 (0.0) | 0 (0) | 0 (0) |  | 0 (0) | 0 (0) | 0 (0) | -- |
| **Peaches** | 33 | 32 | 29 | 0.87 | 662 (65) | 589 (70.5) | 349 (81.4) | 0.99 | 357 (35) | 246 (29.5) | 80 (18.6) | 0.12 | 334 (32.8) | 231 (27.7) | 76 (17.7) | 0.90 | 23 (2.3) | 15 (1.8) | 4 (0.9) | 0.84 |
| **Pear** | 27 | 17 | 17 | 0.87 | 1250 (80.5) | 820 (80.8) | 414 (81.2) | 1.00 | 302 (19.5) | 195 (19.2) | 96 (18.8) | 0.08 | 295 (19.0) | 192 (18.9) | 96 (18.8) | 1.00 | 7 (0.5) | 3 (0.3) | 0 (0) | 0.79 |
| **Perch** | 0 | 0 | 0 |  | 3 (100) | 2 (100) | 1 (100) | 1.00 | 0 (0) | 0 (0) | 0 (0) | -- | 0 (0.0) | 0 (0) | 0 (0) |  | 0 (0) | 0 (0) | 0 (0) | -- |
| **persimmon** | 11 | 4 | 10 | 0.52 | 386 (92.3) | 257 (94.1) | 113 (91.9) | 1.00 | 32 (7.7) | 16 (5.9) | 10 (8.1) | 0.87 | 25 (6.0) | 12 (4.4) | 7 (5.7) | 0.97 | 7 (1.7) | 4 (1.5) | 3 (2.4) | 0.95 |
| **Pineapple** | 12 | 10 | 4 | 0.98 | 824 (93.5) | 541 (93.9) | 304 (97.1) | 1.00 | 57 (6.5) | 35 (6.1) | 9 (2.9) | 0.27 | 49 (5.6) | 29 (5) | 5 (1.6) | 0.66 | 8 (0.9) | 6 (1) | 4 (1.3) | 0.98 |
| **Plum** | 21 | 15 | 12 | 0.97 | 1454 (92.3) | 910 (93.3) | 566 (92.5) | 1.00 | 121 (7.7) | 65 (6.7) | 46 (7.5) | 0.90 | 115 (7.3) | 65 (6.7) | 43 (7) | 1.00 | 6 (0.4) | 0 (0) | 3 (0.5) | 0.87 |
| **Pomegranate** | 22 | 19 | 7 | 0.96 | 1739 (94.9) | 1141 (94.8) | 648 (98) | 1.00 | 94 (5.1) | 62 (5.2) | 13 (2) | 0.34 | 49 (2.7) | 29 (2.4) | 8 (1.2) | 0.84 | 45 (2.5) | 33 (2.7) | 5 (0.8) | 0.64 |
| **Pomelo** | 0 | 0 | 0 |  | 7 (100) | 5 (100) | 2 (100) | 1.00 | 0 (0) | 0 (0) | 0 (0) | -- | 0 (0.0) | 0 (0) | 0 (0) |  | 0 (0) | 0 (0) | 0 (0) | -- |
| **Strawberry** | 40 | 28 | 20 | 0.91 | 764 (75.9) | 541 (75.6) | 265 (88.6) | 0.99 | 243 (24.1) | 175 (24.4) | 34 (11.4) | 0.35 | 212 (21.1) | 144 (20.1) | 30 (10) | 0.84 | 31 (3.1) | 31 (4.3) | 4 (1.3) | 0.73 |
| **Watermelon** | 8 | 8 | 1 | 0.90 | 130 (90.9) | 62 (69.7) | 23 (95.8) | 0.97 | 13 (9.1) | 27 (30.3) | 1 (4.2) | 0.89 | 13 (9.1) | 27 (30.3) | 1 (4.2) | 0.48 | 0 (0) | 0 (0) | 0 (0) | -- |

Note: Data presented as Mean ± SE for overall; N for Pesticide Type and N (%) for Residue Free, With Residue, Residue < MRL and Residue > MRL; P-value obtained from Negative Binomial Regression with Log Link; P-value < 0.05 considered significant.

| **Supplementary Table 2: Instrumental parameters for pesticide residue analysis by LC-MS/MS and GC-MS/MS** | | | | | | | | |
| --- | --- | --- | --- | --- | --- | --- | --- | --- |
| **Pesticide compound** | **Instrument** | **Retention Time**  **(min)** | **Qualifier ion (1)**  **(m/z)** | **Qualifier ion (2)**  **(m/z)** | **Qualifier ion (3)**  **(m/z)** | **Collision Energy (1)**  **(eV)** | **Collision Energy (2)**  **(eV)** | **Collision Energy (3)**  **(eV)** |
| Acetamiprid | **LC-MS/MS** | 4.602 | 223.1>126.1 | 223.1>55.95 | 223.1>73 | -11 | -16 | -55 |
| Carbetamide |  | 4.334 | 237.1>192.1 | 237.1>118.15 | 237.1>120.1 | -5 | -7 | -11 |
| Carbofuran-3-hydroxy (3-Hydroxycarbofuran) |  | 3.947 | 255>163.15 | 255>220.05 | 255>238.05 | -19 | -11 | -8 |
| Clothianidin |  | 3.783 | 250>132.05 | 250>169.1 | 250>113.15 | -16 | -13 | -26 |
| Dimethoate |  | 4.043 | 230>125 | 230>198.9 | 230>171 | -11 | -4 | -14 |
| Ethofumesate |  | 6.645 | 304.1>287 | 304.1>121.1 | 304.1>240.95 | -11 | -22 | -14 |
| Fenhexamid |  | 6.486 | 302.1>97.1 | 302.1>55.05 | 302.1>143 | -24 | -41 | -34 |
| Fenpropimorph |  | 7.617 | 304.2>147.1 | 304.2>117 | 304.2>132.05 | -19 | -55 | -42 |
| Imazali |  | 7.573 | 297.1>159.05 | 297.1>41.1 | 297.1>200.85 | -23 | -31 | -19 |
| Kresoxim-methyl |  | 8.071 | 314.1>267 | 314.1>116.15 | 314.1>235 | -8 | -22 | -16 |
| Methomyl |  | 3.65 | 163>87.9 | 163>106.15 | 163>58 | -10 | -11 | -22 |
| Monocrotophos |  | 3.511 | 240.9>127.1 | 240.9>193 | 240.9>224 | -21 | -12 | -7 |
| Omethoate |  | 2.916 | 214.1>125 | 214.1>183 | 214.1>155 | -18 | -11 | -15 |
| Propiconazole |  | 7.934 | 342>158.9 | 342>69.1 | 342>41 | -28 | -22 | -39 |
| Spirodiclofen |  | 9.415 | 411.1>313.05 | 411.1>71.1 | 411.1>43.05 | -14 | -22 | -50 |
| Propargite |  | 9.235 | 368.2>231.1 | 368.2>175.1 | 368.2>57 | -6 | -16 | -23 |
| 2-Phenylphenol | **GC-MS/MS** | 6.506 | 170.10>141.10 | 141.10>115.10 | 170.10>115.10 | 24 | 18 | 28 |
| Allethrin-3,4 (Bioallethrin) |  | 12.099 | 123.10>81.10 | 136.10>93.10 | 123.10>95.10 | 10 | 14 | 8 |
| Azinphos-ethyl |  | 16.796 | 132.10>77.00 | 160.10>132.10 | 160.10>77.00 | 14 | 4 | 18 |
| Bifenthrin |  | 15.503 | 181.10>166.10 | 181.10>179.10 | 181.10>153.10 | 12 | 12 | 8 |
| Captafol |  | 15.056 | 79.00>77.00 | 79.00>51.00 | 183.10>79.00 | 14 | 20 | 18 |
| Chlorpyrifos |  | 11.152 | 196.90>168.90 | 313.90>257.90 | 313.90>285.90 | 14 | 14 | 8 |
| cis-Permethrine |  | 17.288 | 183.10>153.10 | 183.10>168.10 | 183.10>165.10 | 14 | 14 | 10 |
| Cyfluthrin |  | 17.826 | 163.10>127.10 | 163.10>91.00 | 226.10>206.10 | 6 | 14 | 14 |
| Cypermethrin |  | 18.151 | 163.10>127.10 | 163.10>91.00 | 181.10>152.10 | 6 | 14 | 22 |
| Deltamethrin |  | 19.781 | 180.90>151.90 | 252.90>93.00 | 252.90>171.90 | 22 | 20 | 8 |
| Fenvalerate |  | 19.043 | 225.10>119.10 | 225.10>147.10 | 419.10>225.10 | 20 | 10 | 6 |
| Fipronil |  | 11.874 | 366.90>212.90 | 368.90>214.90 | 366.90>254.90 | 30 | 30 | 22 |
| Fludioxonil |  | 12.905 | 248.00>127.00 | 248.00>154.00 | 182.00>127.00 | 26 | 20 | 16 |
| Fluridone |  | 18.678 | 328.10>259.00 | 328.10>313.00 | 328.10>127.00 | 24 | 22 | 24 |
| Flutolanil |  | 12.848 | 173.00>145.00 | 173.00>95.00 | 281.10>173.00 | 14 | 26 | 12 |
| Prochloraz |  | 17.483 | 180.10>138.10 | 180.10>69.00 | 180.10>95.00 | 12 | 20 | 20 |
| Profenofos |  | 13.015 | 338.90>268.90 | 336.90>266.90 | 338.90>310.90 | 18 | 14 | 6 |
| Pyridaben |  | 17.442 | 147.10>117.10 | 147.10>132.10 | 147.10>119.10 | 22 | 14 | 10 |
| Pyrimethanil |  | 9.37 | 198.10>183.10 | 198.10>118.10 | 198.10>158.10 | 14 | 28 | 18 |
| Quinalphos |  | 12.108 | 146.10>118.00 | 146.10>91.00 | 157.10>129.00 | 10 | 24 | 14 |
| Resmethrin-1 |  | 14.937 | 143.10>128.10 | 171.10>143.10 | 171.10>128.10 | 10 | 6 | 12 |
| trans-Permethrine |  | 17.402 | 183.10>153.10 | 163.10>127.10 | 183.10>168.10 | 14 | 6 | 14 |
| Triadimenol-1 |  | 12.175 | 168.10>70.00 | 128.10>65.00 | 128.10>100.10 | 10 | 22 | 14 |

| **Supplementary Table 3: Validation Data for the Pesticide Residue analysis in fruit samples** | | | | | | |
| --- | --- | --- | --- | --- | --- | --- |
| **pesticide compound** | **Instrument** | **Matrix** | **Linearity (R²)** | **LOQ (mg/kg)** | **Mean (Recovery%) ± SD** | **RSD %** |
| Acetamiprid | **LC-MS/MS** | Cucumber | 0.998 | 0.01 | 90.3 ± 2.0 | 2.2 |
| Carbetamide |  | Cucumber | 0.999 | 0.005 | 96.9 ± 4.4 | 4.5 |
| Carbofuran-3-hydroxy (3-Hydroxycarbofuran) |  | Cucumber | 0.997 | 0.005 | 110.8 ± 6.7 | 6 |
| Clothianidin |  | Cucumber | 0.998 | 0.02 | 111.3 ± 3.4 | 3 |
| Dimethoate |  | Cucumber | 0.999 | 0.005 | 99.2 ± 5.2 | 5.2 |
| Ethofumesate |  | Cucumber | 0.999 | 0.005 | 77.5 ± 4.0 | 5.2 |
| Fenhexamid |  | Grapes | 0.999 | 0.01 | 84 ± 3.1 | 3.7 |
| Fenpropimorph |  | Cucumber | 0.997 | 0.005 | 86.9 ± 2.9 | 3.4 |
| Imazali |  | Cucumber | 0.999 | 0.005 | 86.3 ± 3.9 | 4.5 |
| Kresoxim-methyl |  | Cucumber | 0.997 | 0.015 | 108.9 ± 4.0 | 4.3 |
| Methomyl |  | Cucumber | 0.999 | 0.005 | 108.6 ± 5.8 | 5.4 |
| Monocrotophos |  | Cucumber | 0.998 | 0.005 | 115.8 ± 2.8 | 2.4 |
| Omethoate |  | Cucumber | 0.997 | 0.005 | 112.7 ± 3.2 | 2.8 |
| Propiconazole |  | Cucumber | 0.999 | 0.005 | 102.1 ±7.5 | 7.3 |
| Spirodiclofen |  | Cucumber | 0.999 | 0.005 | 105.2 ± 5.8 | 4.5 |
| Propargite |  | Cucumber | 0.999 | 0.01 | 95 ± 2.3 | 8 |
| 2-Phenylphenol | **GC-MS/MS** | Cucumber | 0.9994 | 0.01 | 111.4 ± 3.8 | 3.5 |
| Allethrin-3,4 (Bioallethrin) |  | Cucumber | 0.999 | 0.1 | 97.5 ± 6.9 | 7.1 |
| Azinphos-ethyl |  | Cucumber | 0.9984 | 0.01 | 82.6 ± 1.7 | 2.2 |
| Bifenthrin |  | Cucumber | 0.9997 | 0.01 | 107.4 ± 3.7 | 3.5 |
| Captafol |  | Grapes | 1 | 0.01 | 87.8 ± 5.4 | 6.1 |
| Chlorpyrifos |  | Grapes | 0.9998 | 0.03 | 81.2 ± 3.87 | 4.8 |
| cis-Permethrine |  | Grapes | 1 | 0.03 | 105.4 ± 5.52 | 5.2 |
| Cyfluthrin |  | Grapes | 0.9999 | 0.06 | 94.4 ± 9.35 | 9.9 |
| Cypermethrin |  | Grapes | 0.9999 | 0.04 | 97.6 ± 9.73 | 11 |
| Deltamethrin |  | Grapes | 0.9997 | 0.06 | 87.9 ± 9.06 | 10.3 |
| Fenvalerate |  | Grapes | 0.9996 | 0.07 | 91.2 ± 9.84 | 12.7 |
| Fipronil |  | Cucumber | 0.9994 | 0.015 | 79.6 ± 5.5 | 6.9 |
| Fludioxonil |  | Cucumber | 0.9989 | 0.05 | 77.1 ± 3.8 | 4.9 |
| Fluridone |  | Cucumber | 0.9972 | 0.1 | 84.2 ± 3.9 | 5.3 |
| Flutolanil |  | Cucumber | 0.9996 | 0.01 | 105.2 ± 4.2 | 4.1 |
| Prochloraz |  | Grapes | 1 | 0.07 | 98.1 ± 4.7 | 4.9 |
| Profenofos |  | Grapes | 0.9999 | 0.1 | 87.8 ± 9.1 | 10.5 |
| Pyridaben |  | Cucumber | 0.9998 | 0.01 | 111.6 ± 2.7 | 2.1 |
| Pyrimethanil |  | Grapes | 0.9992 | 0.01 | 96.6 ± 8.3 | 8.6 |
| Quinalphos |  | Grapes | 0.9999 | 0.04 | 87.5 ± 6.6 | 7.3 |
| Resmethrin-1 |  | Cucumber | 0.9997 | 0.015 | 90.6 ± 7.0 | 7.7 |
| trans-Permethrine |  | Grapes | 0.9999 | 0.08 | 94.5 ± 4.0 | 6.3 |
| Triadimenol-1 |  | Cucumber | 0.9985 | 0.015 | 109.0 ± 3.8 | 3.5 |

**Supplementary Figure 1:** An example of contaminated fruit analysed by GC-MS/MS (kiwi, Chlorpyrifos: 0.0982 mg/kg).

**Supplementary Figure 2:** An example of Chlorpyrifos calibration at GC-MS/MS (R^2^ =0.999, RSD% 4.9)

**Supplementary Figure 3:** An example of contaminated fruit analysed by LC-MS/MS (Apple, Acetamiprid 0.040 mg/kg.

**Supplementary Figure 4:** An example of Acetamiprid Calibration at LC-MS/MS (R^2^ = 0.999, RSD% 5.7)
